# Natural ghrelin in advanced cancer patients with cachexia, a case series

**DOI:** 10.1002/jcsm.12659

**Published:** 2021-01-15

**Authors:** David Blum, Susanne de Wolf‐Linder, Rolf Oberholzer, Michael Brändle, Thomas Hundsberger, Florian Strasser

**Affiliations:** ^1^ Oncological Palliative Medicine, Clinic Oncology/Hematology Cantonal Hospital St. Gallen St. Gallen Switzerland; ^2^ Competence Center Palliative Care, Department of Radiation Oncology Universitätsspital Zürich Zürich Switzerland; ^3^ School of Health Professions, Institute of Nursing Zurich University of Applied Sciences Winterthur Switzerland; ^4^ Wolfson Palliative Care Research Centre, Hull York Medical School Hull University Hull UK; ^5^ Internal Medicine Cantonal Hospital St. Gallen St. Gallen Switzerland; ^6^ Neurology Cantonal Hospital St. Gallen St. Gallen Switzerland

**Keywords:** Ghrelin, Cancer cachexia, Appetite, Muscle mass

## Abstract

**Background:**

Natural ghrelin, a peptide growth hormone secretagogue, has a therapeutic potential in cachexia. We designed a dose‐finding trial of subcutaneous natural ghrelin to improve nutritional intake (NI) in advanced cancer patients.

**Methods:**

Advanced cancer patients with cachexia management (symptom management, physiotherapy, nutritional, and psychosocial support) started with ghrelin at 32 μg/kg body weight, followed by 50% dose increases. Patients self‐injected ghrelin twice daily for 4 days followed by a wash‐out period. After reaching the primary endpoint, maximal NI (minimal dose for maximal NI), a maintenance period followed during which patients injected 10 doses of ghrelin per week. Safety parameters, NI, and cachexia outcomes (symptoms, narratives, muscle mass, and strength) were measured over 6 weeks.

**Results:**

Ten patients with metastatic solid tumours were included, and six (100% male, mean age 61.8 ± 8.5 SD) received ghrelin. Minimal dose for maximal NI was reached in four patients. Three patients reached the end‐of study visit. Ghrelin was well tolerated with variable results on appetite and eating‐related symptoms but a positive effect in the narratives. Mean Functional Assessment of Appetite & Cachexia Therapy score was 6.8 points lower at final measurement compared with baseline, *t*(5) = 5.98, *P* < .01. Muscle mass was stable in two patients and increased in one patient, and muscle strength was stable in three patients. Subjective tolerability was high. Patients showed a fluctuating trajectory, and median survival was 88 days (51–412 days).

**Conclusions:**

Ghrelin was safe in advanced patients with cancer cachexia without dose‐limiting toxicity and well tolerated. The intervention was very complex, and the number of patients included was small. There was a positive effect on nutritional intake and patient narratives.

## Background

Cancer cachexia remains a frequent problem in advanced cancer patients and their families.^1^ It ranges from 24% of advanced cancer patients at the time of diagnosis to >80% at the terminal stages.^2^ Cancer cachexia is clinically relevant because it is related to poor performance status, poor tolerance of anti‐neoplastic treatments,^3^ increased pulmonary infections, symptoms such as fatigue/asthenia,^4^ anorexia, early satiety, chronic nausea, shortness of breath, sleep/wake disturbances, malaise, altered body image, family distress,^5^ and ultimately, unnecessary suffering^6^ and shorter survival.^7^

Key components of cachexia are integrated into clinical classification and assessment: (i) anorexia/reduced food intake, (ii) catabolic drive (caused by inflammation and tumour activity), (iii) decreased muscle mass and strength, and (iv) impact of cachexia (psychosocial and physical functioning).^8^ Current treatments of cancer cachexia include both standard management and pharmacological and nutritional interventions. Standard palliative cancer care provides treatment of the so‐called secondary reasons for cachexia and loss of appetite/decrease nutritional intake, such as stomatitis, dysphagia, uncontrolled symptoms, and constipation.^9^

A basic nutritional counselling programme should be in place to treat dietary problems and support conscious control of eating. Nutritional interventions for cancer‐related cachexia, including enteral and parenteral nutrition and nutritional counselling, have limited effect on only a minority of patients and isolated positive outcomes. Physical activity counselling should encourage patients to make minimal physical stimuli to their muscles. Because emotional and social aspects can affect food intake, psycho‐social support should be in place in order to relieve eating related distress of patients and family members. As pharmacological treatments, currently only progestin (effect on appetite, body weight, mainly water, and thromboembolic complications), corticosteroids (short‐term effect of less than a few weeks, then insulin resistance, muscle wasting and increased risk for infections), and prokinetic agents (mainly for patients with early satiety) are available.^10^

Ghrelin, a 28 amino acid peptide discovered in 1999, is an endogenous ligand for the growth hormone secretagogue receptor, displaying dose‐dependent growth hormone‐releasing activity.^11^ Ghrelin is predominantly secreted by gastric endocrine cells. When administered peripherally, it stimulates growth hormone secretion and food intake, triggers a positive energy balance, and produces weight gain through a central mechanism involving hypothalamic neuropeptides. Ghrelin increases during periods of fasting or under conditions associated with negative energy balance such as starvation or anorexia. In contrast, ghrelin levels are low after eating, or with hyperglycaemia, and in obesity.

Ghrelin has had stimulatory effects on appetite and food intake, lean body mass, gastrointestinal motility, and energy metabolism, and it has alleviated cancer chemotherapy‐associated dyspepsia.^4^, ^12^, ^13^ Ghrelin has a possible effect on skeletal muscle mitochondrial function, inflammatory changes, and insulin signalling.^14^

In one pilot study, seven cancer patients had 31% higher energy intake with intravenous ghrelin than with placebo (5 pmol/kg/min for 180 min) with no adverse effects.^13^ A trial on intravenous ghrelin reports good tolerability and safety of single intravenous application of 2 and 8 μg/kg ghrelin i.v.^15^

The aim of this trial is to investigate the effect of individually dose‐optimized (dose escalation) twice‐daily subcutaneous (s.c.) natural ghrelin on safety, toxicity, tolerability, nutritional intake, anorexia, eating‐related symptoms, muscle mass, and strength and physical function in advanced cancer patients.

## Methods

### Patient population

Adult patients were recruited in the outpatient and inpatient services of the Oncology Department at the Cantonal Hospital St. Gallen, Switzerland, specifically from specialized cancer cachexia clinics.

Patients with any type of advanced incurable solid tumour and cancer cachexia were assessed for eligibility. The tumour situation had to be expected to remain stable without rapid progression in the next weeks. Anti‐neoplastic treatment was allowed if it was given continuously, defined as weekly, or biweekly, and if the treatment did not cause more than Grade 1 adverse event (AE) of decreased oral intake and gastrointestinal dysfunction.

Cancer cachexia was defined as the involuntary loss of weight of 2% of total body weight in 2 months or 5% in 6 months and was ongoing in recent weeks. Patients had to be able to eat, defined as no severe structural barriers in the upper gastrointestinal tract and no bowel obstruction. Patients were not allowed to have corticosteroids except for maximum of 2 days for chemotherapy, no progestin therapy within the last 2 weeks, and no anabolic drugs within the last month. Prokinetic medications were allowed, if given in a fixed dose for 2 weeks before study start, and expected to be continued during the trial period.

Laboratory test results must have been within these ranges: absolute neutrophil count ≥1.5 × 10^9^/L, platelet count ≥100 × 10^9^/L, serum creatinine ≤2.0 mg/dL, total bilirubin ≤1.5 mg/dL, and aspartate aminotransferase and alanine aminotransferase ≤2 × upper limit of normal or ≤5 × upper limit of normal if hepatic metastases are present.

Presence of a normal level of consciousness with a mini mental status of ≥25/30 points was required, and the patient had to give informed consent.

### Intervention

After basic cachexia management, the study was divided into a titration period and a maintenance period.

#### Basic cachexia management

Patients were instructed about individual regular physical activity by a physical therapist and received basic psychosocial/spiritual assessment and treatment. Patients had at least one nutritional counselling session by a specialized nutritionist for optimization of nutritional intake (multiple small meals, food preference, etc.) and for the instruction of recording nutritional intake.

Ghrelin was applied by the research staff at the clinic. During the maintenance period, home application was performed. Ghrelin applied at patients' homes was handed out as vials containing powdered ghrelin. The patient stored the vials for a maximum of 1 week in a special box in the freezer.

The starting dose was 32 μg/kg body weight, which has been shown to be safe in human beings. After the first three patients, the starting dose level was increased to Level 2. In the first four dose levels, the dose was increased by 50%. From the fifth dose level onwards, the increase was 25%:

- Dose level 1 = 32 μg/kg
- Dose level 2 = 48 μg/kg
- Dose level 3 = 72 μg/kg
- Dose level 4 = 108 μg/kg
- Dose level 5 = 135 μg/kg
- Dose level 6 = 169 μg/kg
- Dose level 7 = 211 μg/kg

The maximum tolerable dose was set as 20 mg ghrelin (equivalent to 5 mL) due to the high drug volume to be administered s.c.. Volumes of >2.5 mL had to be divided into two syringes, that is, the subject had to be injected twice. The first dose of each dose level was always administered in the clinic for safety reasons.

#### Titration period in hospital

During the titration period, treatment was conducted with twice daily s.c. ghrelin injection for 2 days.

#### Maintenance period in hospital and at home

All patients who reached the maintenance period injected ghrelin twice daily on 21 days (on five out of seven days per week, i.e. Monday to Friday).

## Outcomes

### Nutritional intake

Nutritional intake was measured according to best practice adapted to the individual patient. This was performed using a food diary, photographs, and the use of a digital scale to weigh meals. In addition, the nutritionist called the patients once per day to remind him/her to complete the food diary. The nutritionists estimated the caloric equivalent of food eaten.

#### Definition of minimal dose for maximal nutritional intake

For each individual patient, data were reviewed to establish the lowest ghrelin drug level associated with the maximum caloric nutritional intake in a 24 h period [minimal dose for maximal nutritional intake (MD‐MANI)]. A substantial increase in intake was defined as at least 10% more caloric intake compared with the baseline. Doses were escalated as long as a substantial increase in intake was measured and no toxicity occurred. MD‐MANI was determined when no significant increase was measured anymore or toxicity occurred. At this point, patients were referred to the maintenance period.

### Toxicity and tolerability

The maximal ghrelin dose level was deemed to be one at which there was no Grade 3/4 AE or serious AE (SAE) likely associated with the investigational product. Common toxicity criteria from the time of first drug administration until study termination and 30 days follow‐up were applied. The assessment of any grade AE and SAE included neuropsychologic side effects (like, e.g. dizziness) and injection site reactions and haematological/biochemical alterations. Assessments were performed according to good clinical practice (GCP).

### Anorexia eating‐related symptoms, narratives

Anorexia assessment was performed using the Edmonton Symptom Assessment Scale.^16^ Eating‐related symptoms like appetite, satiety, nausea, abdominal discomfort, and dyspepsia were assessed by the modified German Functional Assessment of Appetite & Cachexia Therapy (FAACT^+^), which includes 15 items in a 4‐point categorical scale. FAACT^+^ is an adapted and translated version of the FAACT questionnaire.^17^ Additionally, narratives from patients and proxies were collected.

### Muscle mass and muscle strength (muscularity)

Muscle mass was measured by abdominal computerized tomography scan. Images were analysed using Slice‐O‐matic software V4.3 (Tomovision) using pre‐established thresholds of Hounsfield unit.^18^, ^19^

At each time point, hand grip strength was measured six times. Compliance of the patient was estimated in order to get reliable test results. The mean value out of all six measures of each hand was calculated. (JAMAR® Hand Dynamometer).^20^

### Physical function

A validated activity monitor the ActivPAL (PALTechnologies Ltd, Glasgow, Scotland), based on a uni‐axial accelerometer was used.^21^ It identifies episodes of walking, sitting, and standing, allowing the measurement of both activity and inactivity. Furthermore, it records the number of steps and instantaneous cadence. Each time span of measurement was 3 days.

### Statistical analyses

All patients were included in the analyses. Descriptive analysis was performed. Paired *t* tests were conducted on pre–post ghrelin measurements. The level of statistical significance was set at *P* < .05.

### Ethical considerations

The study protocol was written and the trial was performed in accordance with the Declaration of Helsinki, the Guidelines of Good Clinical Practice issued by the International Council for Harmonisation of Technical Requirements for Pharmaceuticals for Human Use and Swiss regulatory authority's requirements.

The Study was approved by the local ethics committee (EKSG SG 294/08).

## Results

**FIGURE 1:**
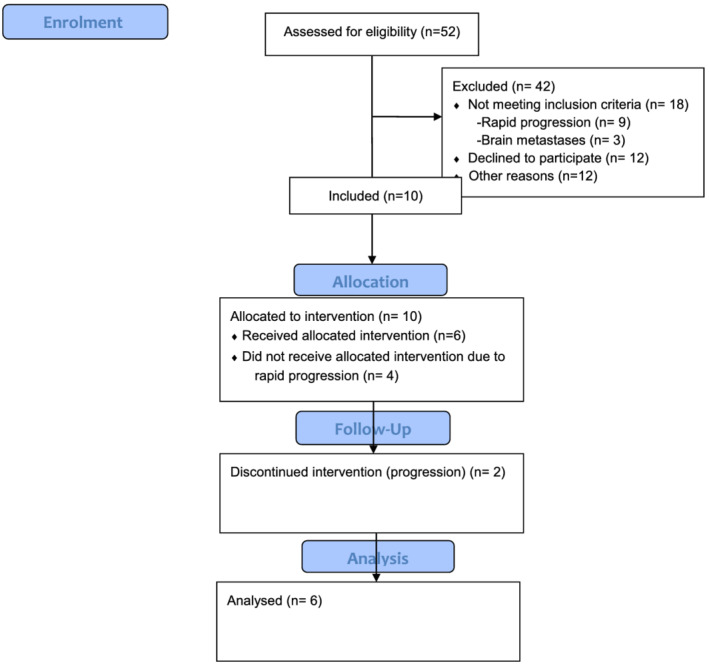
CONSORT flow diagram.

Over a 1 year period, patients in the cachexia clinics were screened for trial participation. The CONSORT‐diagram is displayed in Figure 1.

Ten patients were included in the study, but only six patients received the investigational product. The remaining four patients never got the drug due to rapid progression of the cancer disease.

Age ranged from 48 to 76 years. The tumour types represented were pancreatic, head and neck, lung, and gastrointestinal cancer. Average weight loss in the last 6 months was 15 kg.

An overview on patient demographics is given in Table 1, and MD‐MANI is presented in Table 2. In the following paragraphs, patients receiving Ghrelin are described case by case:

**Table 1:** Demographics

Patient	Tumour type	Metastasis^1^	Chemotherapy	Age (years)	Sex	Karnofsky	ECOG	Weight (kg)	BMI (kg/m^2^)	Wl2m (kg)	Wl6m (kg)	NI (%)	Trajectory	Survival (days)
1	Pancreas	2, 5	Gemcitabine	61	Male	80	1	75	25,4	−14,7	−16,7	75	PR	412
2	Pancreas	4,5	Gemcitabine	66	Male	70	2	51,6	17,9	0,4	−9,2	15	N/A	53
3	Gastrointestinal	2,5	Capecitabine	76	Male	60	2	48	16,0	−12,5	−14,3	15	N/A	51
5	Mesothelioma	6	Capecitabine Irinotecan	63	Male	60	1	70	22,6	1–1,4	N/A	30	PD	62
9	Head and neck	1,2,4, 5	None	48	m	80	1	35	15,8	−11,4	−16,7	73	PR	114d
10	Head and neck	1,6	Cetuximab	57	m	90	1	59	18,6	3,4	−19,2	85	PD	166d

Metastasis location, 1 = lung; 2 = liver; 3 = brain; 4 = lymph node; 5 = other; 6 = none.

BMI, body mass index; ECOG, Eastern Cooperative Oncology Group; NI, nutritional intake; Wl2m: weight loss 2 months;Wl6m: weight loss 6 months.

**Table 2:** Nutritional intake with MD‐MANI in bold

**Patient 1**	Dose level	1	**2**	3	4	2	2	2
	Kcal	2350	**2691**	2415	2627	2650	2005	2581
	Increase BL (%)	0	**+14**	+3	+12	+12	−15%	−10
**Patient2**	Dose level	1	2	3	4	5	0	0
	Kcal	934	1,394	1,345	999	752	971	1047
	Increase BL (%)	−31.50	2	−1	−26.8	−44	−28	−23
**Patient3**	Dose level	1	2	3	**4**	5	4	0
	Kcal	1061	1189	1285	**1331**	1275	337	246
	Increase BL (%)	−6	+6	+14	**+18**	+13	−70	−78
**Patient 5**	Dose level	2	**3**	4*	5	3	3	3
	Kcal	1580	**1908**	1585	1553	1220	1554	1843
	Increase BL (%)	+7	**+29**	+7	+5	−18	+5	+25
**Patient 9**	Dose level	2	**3**	4	5	3	3	3
	Kcal	3035	**3544**	3044	3194	3404	3125	2559
	Increase BL (%)	−5	**+11**	−5	−0	+6	−35	−20
**Patient 10**	Dose level	2	**3**	4	3	3	3	
	Kcal	1900	**2029**	2027	1913	1863	2322	
	Increase BL (%)	+3	**+11**	+10	+4	+1	+26	

Due to S‐NIS trial, chair decided to rise one level.

BL, baseline; MD‐MANI, minimal dose for maximal nutritional intake.

### Patient 1

A 61‐year‐old male patient with pancreatic cancer, which had metastasized to the liver, presented with a weight of 75 kg and body mass index (BMI) of 25.4. In the previous six months, he had lost 17 kg, 15 kg of which had been lost in the previous two months. He received Gemcitabine. He had a Karnofsky score of 80 and an Eastern Cooperative Oncology Group (ECOG) score of 1. This patient reached MD‐MANI at Dose Level 2 with a 14% increase in kilocalorie intake compared with baseline. Patient 1 commented about his experience using ghrelin saying, ‘I don't leave home without a snack’ and ‘I have to eat every two to three hours.’ Comparing pre‐study measurements with post‐study measurements, he exhibited no change in muscle mass, a 2% increase in muscle strength, and a 7% decrease in steps per day. This patient survived 412 days.

### Patient 2

A 66‐year‐old male patient with pancreatic cancer, which had metastasized to the lymph nodes, presented with a weight of 52 kg and a BMI of 17.9. He had lost nine kg in the previous six months, including 0.4 kg of weight gain in the previous two months. He received Gemcitabine. He had a Karnofsky score of 70 and an ECOG core of 2. MD‐MANI could not be established for this patient. At all ghrelin dose levels, kilocalorie intake was lower than baseline, with the exception of Dose Level 2, when there was a 2% increase in kilocalorie intake. This small increase did not meet the pre‐set definition of MD‐MANI being at least 10% increase in kilocalorie compared to baseline. Patient 2 experienced an SAE, an episode of hypothermia, which was deemed attributable to the ghrelin application. Nonetheless, this patient reported positive impressions of taking ghrelin, stating that ‘I feel fresher and I eat more.’ Over the course of the study, he exhibited a 5% decrease in muscle mass. Post‐study measurements of muscle strength steps per day were not available. This patient survived 53 days.

### Patient 3

A 76‐year‐old male patient with gastrointestinal cancer, which had metastasized to the liver, presented with a weight of 48 kg and a BMI of 16.0. In the previous 6 months, he had lost 14 kg, 13 of which had been lost in the previous 2 months. He received Capecitabine. He had a Karnofsky score of 60 and an ECOG score of 2. This patient reached MD‐MANI at Dose Level 4, when there was an 18% increase in kilocalorie intake compared with baseline. Patient 3 made the following comment about using ghrelin: ‘Feel more active.’ During the study period, Patient 3 was hospitalized for atrial fibrillation, which was an SAE deemed unrelated to ghrelin. This patient survived 51 days.

### Patient 5

A 63‐year‐old male patient with advanced mesothelioma presented with a weight of 70 kg and a BMI of 22.6. Data regarding weight loss in the previous 6 months were not available.. He received FOLFIRI. He had a Karnofsky score of 60 and an ECOG score of 1. This patient reached MD‐MANI at Dose Level 3 with a 29% increase in kilocalorie intake compared with baseline. Patient 5's opinion about using ghrelin was positive, saying ‘This is the first therapy which improves my well‐being.’ Patient 5 experienced an increase in tumour pain, an SAE deemed unrelated to ghrelin. Comparing pre‐study measurements with post‐study measurement, he exhibited a 3% decrease in muscle mass. Post‐study measurements for muscle strength, steps per day could not be obtained. This patient survived 62 days.

### Patient 9

A 48‐year‐old male patient with head and neck cancer, which had metastasized to the lungs, liver, and lymph nodes, presented with weight of 35 kg and a BMI of 15.8. He had lost 17 kg in the previous 6 months, of which 11 kg had occurred in the previous 2 months. He did not receive chemotherapy. He had a Karnofsky score of 80 and an ECOG score of 1. This patient reached MD‐MANI at Dose Level 3, at which point the patient took in 11% more kilocalorie compared with baseline. A secondary malignancy was diagnosed in Patient 9, an SAE deemed unrelated to ghrelin. Comparing pre‐study measurements with post‐study measurements, he exhibited a 9% decrease in muscle mass, a 6% increase in muscle strength, and a 19% decrease in steps per day. This patient survived 114 days.

### Patient 10

A 57‐year‐old male patient with head and neck cancer, which had metastasized to the lungs, presented with a weight of 59 kg and a BMI of 18.6. He had lost 19 kg in the previous 6 months, including 3 kg of weight gain in the previous 2 months. He received Cetuximab. He had a Karnofsky score of 90 and an ECOG score of 1. This patient reached MD‐MANI at Dose Level 3 when kilocalorie intake increased 11% compared with baseline. Patient 10 commented that on ghrelin, ‘my stomach growls’. Comparing pre‐study measurements with post‐study measurements, he exhibited no change in muscle mass, a 6% increase in muscle strength, and a 16% increase in steps per day. This patient survived 166 days.

### Anorexia and eating‐related symptoms

**FIGURE 2:**
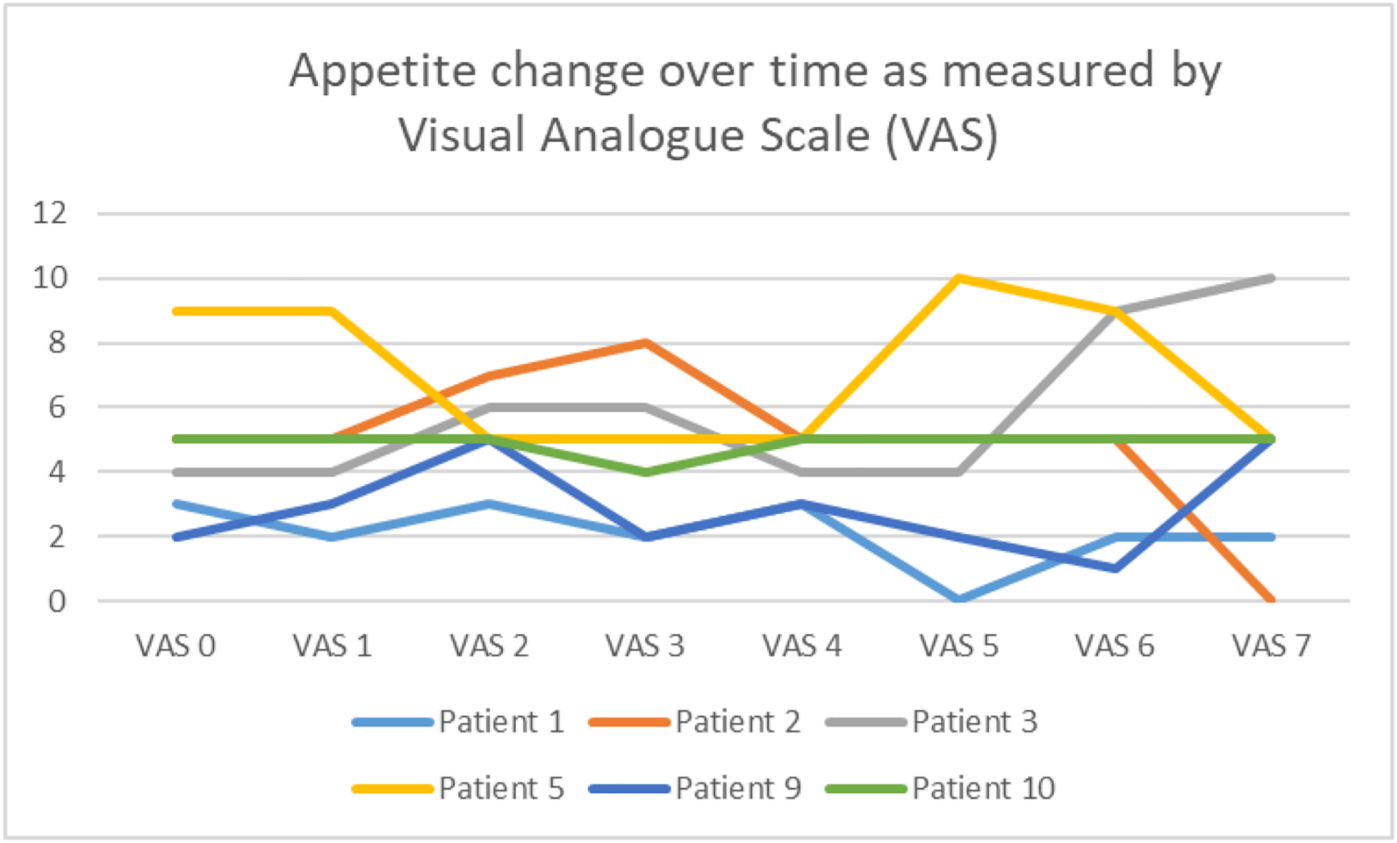
Appetite. Appetite VAS (1–10, 0 *best*, 10 *worst*).

Anorexia, as measured on a visual analogue scale, showed fluctuation during the study as shown in Figure 2. FAACT sum scores trajectories are displayed in Table 3. A paired *t* test showed that mean FAACT scores were 6.8 points lower, significantly lower at the final measurement as compared with the first measurement, *t*(5) = 4.88, *P* = 0.005. A paired *t* test of the first and last visual analogue scale measurements showed no statistically significant change, *t*(5) = −0.47, *P* = 0.67.

**Table 3:** Functional Assessment of Appetite & Cachexia Therapy (FAACT)

	FAACT 0	FAACT 1	FAACT 2	FAACT3	FAACT 4	FAACT 5	FAACT 6	FAACT 7
Patient 1	33	31	31	32	33	33	30	30
Patient 2	38	28	27	27	31	33	N/A	N/A
Patient 3	34	30	33	27	24	26	N/A	N/A
Patient 5	35	35	34	33	34	35	35	22
Patient 9	38	41	41	37	40	37	40	32
Patient 10	38	33	31	30	31	35	32	N/A

FAACT (lower scores better).

N/A, not available.

### Muscle mass and strength, physical activity

Muscle mass was stable in two patients and increased in one patient, and muscle strength was stable in three patients. The full results can be seen in Table 4.

**Table 4:** Muscle mass and strength

	Muscle mass TAG (cm^3^)			Muscle strength (kg)		
	Before	After	% change	Before	After	% change
Patient 1	115	115	0%	28.4	28.9	2%
Patient 2	95	90	−5%	17.8	ND	N/A
Patient 5	155	150	−3%	12.0	ND	N/A
Patient 8	115	125	9%	44.6	43.7	−2%
Patient 9	120	109	−9%	35.0	37	6%
Patient 10	115	115	0%	34.0	36	6%

N/A, not available; ND, no data.

The change in steps walked was also highly variable, depending on the patient. The change in step count from first measurement to last measurement ranged from −92% to 100%. The difficulties of measuring physical activity among a palliative cohort were apparent. For example, Patient 5 was non‐compliant when it came to using the ActivPAL device, due to his perception that the device was inconvenient. Results are displayed in Table 5.

**Table 5:** Daily step counts

Patient number	Date	Daily step count	% Change
1	23/06/09	2210	
1	24/06/09	2828	
1	25/06/09	4418	
1	13/07/09	4796	
1	14/07/09	5596	
1	15/07/09	4956	
1	01/08/09	5616	
1	02/08/09	3294	
1	03/08/09	3938	
1	11/12/09	6948	
1	12/12/09	1806	
1	13/12/09	1896	
1	14/12/09	2056	−7%
2	23/06/09	2210	
2	24/06/09	2828	
2	25/06/09	4418	100%
3	10/08/09	6764	
3	01/09/09	532	−92%
5	—	—	—
9	25/03/10	2886	—
9	26/03/10	3852	—
9	27/03/10	5884	—
9	28/03/10	3836	—
9	29/03/10	1302	—
9	16/04/10	6662	—
9	17/04/10	10 812	—
9	18/04/10	3936	—
9	19/04/10	202	—
9	07/05/09	3234	—
9	08/05/09	6940	—
9	09/05/09	2342	−19%
10	20/07/10	5400	—
10	21/07/10	6748	—
10	22/07/10	3228	—
10	23/07/10	3650	—
10	24/07/10	2340	—
10	27/08/10	7718	—
10	28/08/10	6896	—
10	29/08/10	2740	—
10	30/08/10	13 622	—
10	31/08/10	860	—
10	01/09/10	6290	16%

The injections were well tolerated at the injection site, and subjective tolerability was high. Patients showed a fluctuation trajectory, and survival was short: median survival was 88 days (min 51 days; max 412 days).

The appendix provides additional data about concomitant medications, vital signs at baseline, C‐reactive protien levels, and adverse events.

## Discussion

All six patients who received s.c. ghrelin in the study suffered from far advanced cancer and experienced major weight loss. Five of the six patients were under anticancer treatment. All of them were male. Ghrelin was well tolerated and was perceived positively by individual study participants. There was a positive effect on nutritional intake, and MD‐MANI could be determined in four out of six patients. There seemed to be an effect with titration but without linearity. Overall, a definite dose for maximal nutritional intake could not be decided due to the variability of patient outcomes and the small sample size.

The two best responders went into a compassionate use programme because they did not want to abstain from the drug. This was approved by the ethics committee. There was a change in appetite, and according to patient narratives, eating experience and eating‐related symptoms also improved.

During the trial, four SAEs occurred. A clinical relevant hypothermia was considered to be related to the investigational product.^22^ The other events were considered as not drug related. An occurrence of a secondary malignancy was seen; this was considered as not unusual in a patient heavily pre‐treated with several cytotoxic agents.

Muscle mass and muscle strength measures yielded variable results, and ActivPAL measurement encountered technical challenges with extreme fluctuations in activity between days. More measurements and longer measurement times could have improved the chances of drawing meaningful conclusions. A positive effect on muscle mass is difficult to measure in an advanced cancer population because the natural course of muscle loss is difficult to predict. Additionally, symptoms perception and subjective narrative response is influenced by several factors, such as motivation, stimulation by proxies, and rapport with the treatment team.

In recent years, several trials have been conducted with substances similar to ghrelin. Anamorelin, a synthetic growth hormone secretagogue receptor agonist, has been investigated in chemotherapy trials to increase lean body mass and improve muscle strength. Anamorelin improved muscle mass but not the functional co‐primary functional outcome, handgrip strength.^23^, ^24^ The Food and Drug Administration and the European Medicines Agency declined approval, but ghrelin and analogue substances remain an interesting topic in cachexia research.

The strength of the present case series is the innovative design and the measurement of state‐of‐the‐art outcomes in cachexia research. A major weakness is the low number of patients included. Conducting the study was very laborious, and it was challenging to find stable phases in dynamic trajectories near end of life. Because there was no comparator, the study is prone to placebo effects.

Patients with advanced cancer experienced unexpected tumour progression and presented with heavily fluctuating symptoms, which affected nutritional intake. To reflect the proposed effect of ghrelin on appetite, food intake, muscle, inflammation, and psychological measurements, a multitude of outcomes representing different domains of cancer cachexia were measured, because meaningful outcomes in cancer cachexia research remain a matter of debate.^25^ These target outcomes may change along the trajectory of cancer cachexia.^26^ In the early stage of cachexia, muscle mass and function are the central focus. An effective drug could even prevent the development or progression of cachexia. This is in contrast to the end stage of cachexia, when symptom control and palliation become priorities. In this series, Ghrelin was applied mainly near the end of life, but further study of Ghrelin is compelling because it is one of the few substances that could potentially have a positive effect along the whole cachexia trajectory.

## Conflict of interest

David Blum, Susanne de Wolf‐Linder, Rolf Oberholzer, Michael Brändle, Thomas Hundsberger, and Florian Strasser declare that they have no conflict of interest.

## Funding

Investigator initiated trial, performed by KSSG, study drug (vialing) was supported by Gastrotech Pharma A/S and Bachem.

## Appendix A.

**Table A1:** Concomitant medication

Patient number	Medication name	Group
1	Mephamesone	Steroid
1	Mirtazapine	Antidepressant
1	Esomeprazole	Antacid
1	Domperidone	Prokinetic
1	Laxoberon	Laxans
1	Methylphenidate	Stimulants
1	Esomeprazole	Antacid
1	Domperidone	Prokinetic
1	Laxoberon	Laxans
1	Gemcitabine	Cytotoxic agent
1	Mephamesone	Steroid
2	Erythrocyte concentrate	Blood
2	Mephamesone	Steroid
2	Gemcitabine	Cytotoxic agent
3	Folfiri	Cytotoxic agent
5	Natirii Picosulfas Monohydricum	Laxans
5	Macroolum B350	Laxans
5	Metoclopramide	Prokinetic
5	Cisplatin	Cytotoxic agent
5	Carboplatin	Cytotoxic agent
5	Pemetrexed	Cytotoxic agent
9	Dalteparin	Heparin
9	Cetuximab	EGFR inhibitor
10	Mistletoe	Complementary

EGFR, estimated glomerular filtration rate.

**Table A2:** Vital signs on baseline day

Patient number	BP systolic mmHg (Baseline day)	BP diastolic mmHg (Baseline day)	Pulse: beats/min (Baseline day)	Temperature (°C)
1	103	75	84	36.4
2	92	55	71	36.6
3	106	69	79	36.0
5	139	85	80	—
9	125	80	104	36.2
10	107	69	72	36.8

**Table A3:** C‐reactive protein

Patient number	Measurement event	CRP (mg/L)
1	Baseline	12
1	Titration 1	38
1	Titration 2	8
1	Titration 3	23
1	Titration 4	11
1	Maintenance 1	8
1	Maintenance 2	54
1	Maintenance 3	12
1	Conclusion	18
2	Baseline	120
2	Titration 1	221
2	Titration 2	226
2	Titration 3	187
2	Titration 4	157
2	Titration 5	158
2	SAE	144
2	Conclusion	147
3	Baseline	103
3	Titration 1	186
3	Titration 2	119
3	Titration 3	133
3	Titration 4	146
3	Maintenance 1	98
5	Baseline	169
5	Titration 1	150
5	Titration 2	132
5	Titration 3	122
5	Titration 4	134
5	Maintenance 1	134
5	Maintenance 2	125
5	Maintenance 3	113
5	Conclusion	104
9	Baseline	13
9	Titration 1	10
9	Titration 2	23
9	Titration 3	14
9	Titration 4	6
9	Maintenance 1	18
9	Maintenance 2	23
9	Maintenance 3	43
9	Conclusion	61
10	Baseline	9
10	Titration 1	8
10	Titration 2	11
10	Titration 3	19
10	Maintenance 1	17
10	Maintenance 2	9
10	Maintenance 3	110
10	Conclusion	18

**Table A4:** Adverse events

Patient number	Adverse event	Date	Grade according to CTCAEv.3.0	Relationship (2 = *unlikely related*; 3 = *possibly related*; 4 = *probably related*; 5 = *definitely related*)	Time (days) from last ghrelin application (and dose) to AE	Outcome (1 = *recovered*; 2 = *recovering*; 3 = *not recovered*; 4 = *recovered with sequelae*)
1	Constitutional symptoms—other—starving attack	06/07/09–21/07/09	1	3	Titration/3 days/Level 2: 36 μg/kg	1
1	Gastrointestinal—diarrhoea	06/07/09–22/07/09	1	3	Titration/3 days/Level 2: 36 μg/kg	1
1	Gastrointestinal—anorexia	16/07/09–19/07/09	1	3	Maintenance/last BD on the 16/07/09/Level 2: 36 μg/kg	3
1	Gastrointestinal—distension/bloating	18/07/09–19/07/09	1	3	Maintenance/1 day/Level 2: 36 μg/kg	2
1	Endocrine—diabetes (pancreatic endocrine: gluc. intolerance)	20/07/09–nk	3	3	Maintenance week 2/2 days/Level 2: 36 μg/kg	3
2	Constitutional symptoms—hypothermia	10/07/09–10/07/09	2	5	Titration/15 min/Level 5: 135 μg/kg	1
2	Endocrine—flushes	10/07/09–10/07/09	1	5	Titration/15 min/Level 5: 135 μg/kg	1
2	Cardiac arrhythmia—sinus bradycardia	10/07/09–10/07/09	1	5	Titration/15 min/Level 5: 135 μg/kg	1
3	Atrial fibrillation	07/03/09–07/03/09	2	2	unavailable	1
5	Gastrointestinal—diarrhoea	30/11/09–06/12/09	1	3	Titration/2 days/Level 5: 135 μg/kg	1
5	Increased tumour pain	30/11/09–06/12/09	1	2	Unavailable	1
9	Pain—musculoskeletal—muscle—upper left arm	19/05/10–15/06/10	2	3	Maintenance 3/14 days/Level 3: 72 μg/kg	1
9	Secondary malignancy—sarcoma; DD undiff/ squamous cell carcinoma	20/05/10–N/A	3	3	Maintenance 3/15 days/Level 3: 72 μg/kg	4
10	Cardiac general—hypotension	29/07/10–02/08/10	1	4	Titration/90 min/Level 3: 72 μg/kg	1

BD, baseline day; N/A, not available.

## References

[1] Strasser F , Bruera ED . Update on anorexia and cachexia. Hematol Oncol Clin North Am 2002;16:589–617. 12170570 10.1016/s0889-8588(02)00011-4

[2] Dewys WD , Begg C , Lavin PT , Band PR , Bennett JM , Bertino JR , et al. Prognostic effect of weight loss prior to chemotherapy in cancer patients. Am J Med 1980;69:491–497. 7424938 10.1016/s0149-2918(05)80001-3

[3] Andreyev HJN , Norman AR , Oates J , Cunningham D . Why do patients with weight loss have a worse outcome when undergoing chemotherapy for gastrointestinal malignancies? Eur J Cancer 1998;34:503–509. 9713300 10.1016/s0959-8049(97)10090-9

[4] Radbruch L , Strasser F , Elsner F , Gonçalves JF , Løge J , Kaasa S , et al. Fatigue in palliative care patients—an EAPC approach. Palliat Med 2008;22:13–32. 18216074 10.1177/0269216307085183

[5] Strasser F , Binswanger J , Cerny T , Kesselring A . Fighting a losing battle: eating‐related distress of men with advanced cancer and their female partners. A mixed‐methods study. Palliat Med 2007;21:129–137. 17344261 10.1177/0269216307076346

[6] Walsh D , Donnelly S , Rybicki L . The symptoms of advanced cancer: relationship to age, gender, and performance status in 1,000 patients. Support Care Cancer 2000;8:175–179. 10789956 10.1007/s005200050281

[7] Blum D , Stene GB , Solheim TS , Fayers P , Hjermstad MJ , Baracos VE , et al. Validation of the consensus‐definition for cancer cachexia and evaluation of a classification model—a study based on data from an international multicentre project (EPCRC‐CSA). Ann Oncol 2014;25:1635–1642. 24562443 10.1093/annonc/mdu086

[8] Fearon K , Strasser F , Anker SD , Bosaeus I , Bruera E , Fainsinger RL , et al. Definition and classification of cancer cachexia: an international consensus. Lancet Oncol 2011;12:489–495. 21296615 10.1016/S1470-2045(10)70218-7

[9] Omlin A , Blum D , Wierecky J , Haile SR , Ottery FD , Strasser F . Nutrition impact symptoms in advanced cancer patients: frequency and specific interventions, a case‐control study. J Cachexia Sarcopenia Muscle 2013;4:55–61. 23307589 10.1007/s13539-012-0099-x PMC3581613

[10] Yavuzsen T , Davis MP , Walsh D , LeGrand S , Lagman R . Systematic review of the treatment of cancer‐associated anorexia and weight loss. J Clin Oncol 2005;23:8500–8511. 16293879 10.1200/JCO.2005.01.8010

[11] Kojima M , Hosoda H , Date Y , Nakazato M , Matsuo H , Kangawa K . Ghrelin is a growth‐hormone‐releasing acylated peptide from stomach. Nature 1999;402:656–660. 10604470 10.1038/45230

[12] Liu YL , Malik NM , Sanger GJ , Andrews PL . Ghrelin alleviates cancer chemotherapy‐associated dyspepsia in rodents. Cancer Chemother Pharmacol 2006;58:326–333. 16435157 10.1007/s00280-005-0179-0

[13] Neary NM , Small CJ , Wren AM , Lee JL , Druce MR , Palmieri C , et al. Ghrelin increases energy intake in cancer patients with impaired appetite: acute, randomized, placebo‐controlled trial. J Clin Endocrinol Metabol 2004;89:2832–2836. 10.1210/jc.2003-031768 15181065

[14] Barazzoni R , Gortan Cappellari G , Palus S , Vinci P , Ruozi G , Zanetti M , et al. Acylated ghrelin treatment normalizes skeletal muscle mitochondrial oxidative capacity and AKT phosphorylation in rat chronic heart failure. J Cachexia Sarcopenia Muscle 2017;8:991–998. 29098797 10.1002/jcsm.12254 PMC5700435

[15] Strasser F , Lutz TA , Maeder MT , Thuerlimann B , Bueche D , Tschöp M , et al. Safety, tolerability and pharmacokinetics of intravenous ghrelin for cancer‐related anorexia/cachexia: a randomised, placebo‐controlled, double‐blind, double‐crossover study. Br J Cancer 2008;98:300–308. 18182992 10.1038/sj.bjc.6604148 PMC2361459

[16] Bruera E , Kuehn N , Miller MJ , Selmser P , Macmillan K . The Edmonton Symptom Assessment System (ESAS): a simple method for the assessment of palliative care patients. J Palliat Care 1991;7:6–9. 1714502

[17] Cella DF , Tulsky DS , Gray G , Sarafian B , Linn E , Bonomi A , et al. The Functional Assessment of Cancer Therapy scale: development and validation of the general measure. J Clin Oncol 1993;11:570–579. 8445433 10.1200/JCO.1993.11.3.570

[18] Mourtzakis M , Prado CM , Lieffers JR , Reiman T , McCargar LJ , Baracos VE . A practical and precise approach to quantification of body composition in cancer patients using computed tomography images acquired during routine care. Appl Physiol Nutr Metab 2008;33:997–1006. 18923576 10.1139/H08-075

[19] Shen W , Punyanitya M , Wang Z , Gallagher D , St.‐Onge MP , Albu J , et al. Total body skeletal muscle and adipose tissue volumes: estimation from a single abdominal cross‐sectional image. J Appl Physiol (1985) 2004;97:2333–2338. 15310748 10.1152/japplphysiol.00744.2004

[20] Kilgour RD , Vigano A , Trutschnigg B , Lucar E , Borod M , Morais JA . Handgrip strength predicts survival and is associated with markers of clinical and functional outcomes in advanced cancer patients. Support Care Cancer 2013;21:3261–3270. 23872952 10.1007/s00520-013-1894-4

[21] Dahele M , Skipworth RJ , Wall L , Voss A , Preston T , Fearon KC . Objective physical activity and self‐reported quality of life in patients receiving palliative chemotherapy. J Pain Symptom Manage 2007;33:676–685. 17360150 10.1016/j.jpainsymman.2006.09.024

[22] Wiedmer P , Strasser F , Horvath TL , Blum D , DiMarchi R , Lutz T , et al. Ghrelin‐induced hypothermia: a physiological basis but no clinical risk. Physiol Behav 2011;105:43–51. 21513721 10.1016/j.physbeh.2011.03.027 PMC3146973

[23] Temel JS , Abernethy AP , Currow DC , Friend J , Duus EM , Yan Y , et al. Anamorelin in patients with non‐small‐cell lung cancer and cachexia (ROMANA 1 and ROMANA 2): results from two randomised, double‐blind, phase 3 trials. Lancet Oncol 2016;17:519–531. 26906526 10.1016/S1470-2045(15)00558-6

[24] Takayama K , Katakami N , Yokoyama T , Atagi S , Yoshimori K , Kagamu H , et al. Anamorelin (ONO‐7643) in Japanese patients with non‐small cell lung cancer and cachexia: results of a randomized phase 2 trial. Support Care Cancer 2016;24:3495–3505. 27005463 10.1007/s00520-016-3144-z PMC4917578

[25] Fearon K , Argiles JM , Baracos VE , Bernabei R , Coats AJ , Crawford J , et al. Request for regulatory guidance for cancer cachexia intervention trials. J Cachexia Sarcopenia Muscle 2015;6:272–274. 26675232 10.1002/jcsm.12083 PMC4670733

[26] Laird BJA , Balstad TR , Solheim TS . Endpoints in clinical trials in cancer cachexia: where to start? Curr Opin Support Palliat Care 2018;12:445–452. 30299325 10.1097/SPC.0000000000000387

[27] von Haehling S , Morley JE , Coats AJ , Anker SD . Ethical guidelines for publishing in the *Journal of Cachexia, Sarcopenia and Muscle*: update 2019. J Cachexia Sarcopenia Muscle 2019;10:1143–1145. 31661195 10.1002/jcsm.12501 PMC6818444

